# GalNT2-mediated O-glycosylation affects pancreas development and function in mice

**DOI:** 10.1038/s41598-024-80276-7

**Published:** 2024-11-30

**Authors:** Baris Mercanoglu, Sissy-Alina Waschkowski, Elena Neuburg, Nina Schraps, Anastasios D. Giannou, Benjamin Dreyer, Sönke Harder, Markus Heine, Christian F. Krebs, Cenap Güngör, Hartmut Schlüter, Nathaniel Melling, Thilo Hackert, Maximilian Bockhorn, Christoph Wagener, Gerrit Wolters-Eisfeld

**Affiliations:** 1 Department of General, Visceral and Thoracic Surgery, University Medical Center Hamburg-Eppendorf, 20246 Hamburg, Germany; 2 Department of Dermatology and Allergy, University Hospital Schleswig-Holstein, Kiel, Germany; 3 Section of Molecular Immunology und Gastroenterology, I. Department of Medicine, University Medical Center Hamburg-Eppendorf, Hamburg, Germany; 4 Mass Spectrometric Proteomics - Institute for Clinical Chemistry & Laboratory Medicine, University Medical Center Hamburg-Eppendorf, Hamburg, Germany; 5 Department of Biochemistry and Molecular Cell Biology, University Medical Center Hamburg-Eppendorf, Hamburg, Germany; 6 III. Department of Medicine, University Medical Center Hamburg-Eppendorf, Hamburg, Germany; 7 Hamburg Center for Translational Immunology (HCTI), University Medical Center Hamburg-Eppendorf, Hamburg, Germany; 8 Department of General and Visceral Surgery, University Medical Center Oldenburg, 26133 Oldenburg, Germany; 9 Medical Faculty, Universität Hamburg, 20246 Hamburg, Germany

**Keywords:** GalNT2, O-glycosylation, Pancreas, Atrophy, Pancreatic steatosis, Cell biology, Glycobiology, Gastrointestinal models

## Abstract

GALNT2, also known as polypeptide N-acetylgalactosaminyltransferase 2, is an enzyme that catalyzes the initial step of O-linked glycosylation, a crucial posttranslational modification that affects protein folding, stability, and function. Alterations in GALNT2 activity have been implicated in various diseases, such as cancer, metabolic disorders, and cardiovascular diseases, highlighting its importance in maintaining normal physiological functions. To investigate the impact of GalNT2 overexpression in vivo for the first time, we generated a conditional transgenic mouse line in which GalNT2 was expressed specifically in the pancreas. Heterozygous overexpression leads to a loss of acinar mass and pancreatic steatosis, whereas homozygous overexpression causes complete pancreatic loss and results in a lethal phenotype. Using a reporter gene mouse line, we demonstrated that adipocytes originate through transdifferentiation from pancreatic cells. GalNT2 overexpression results in additional O-glycosylation sites, which we analyzed through PNA lectin enrichment and mass spectrometric proteome analysis.

**Supplementary Information:**

The online version contains supplementary material available at 10.1038/s41598-024-80276-7.

## Introduction

The intricate landscape of glycosylation generates a vast array of glycan moieties, glycoproteins, and glycolipids, thereby introducing a dynamic and adjustable layer of regulation for intra- and intercellular signaling. GalNT2 (polypeptide N-acetylgalactosaminyltransferase 2) is part of the GalNAc-T enzyme family^1^, which initiates O-linked glycosylation^2^. This family of glycosyltransferases catalyzes the addition of N-acetylgalactosamine (GalNAc) to serine, threonine, or tyrosine residues^3^on polypeptides, a critical step in the formation of mucin-like O-glycans^4^. This pathway is active primarily in the densely clustered, heavily glycosylated regions of mucins and glycoproteins with mucin-like domains. However, many other proteins contain isolated sites of GalNAc-O-glycosylation^5,6^. To date, more than 2,300 human proteins involved in the secretory pathway have been identified as containing one or more mucin-type O-glycans^2,7^.

GALNT2 is ubiquitously expressed and localized in the Golgi apparatus, where it interacts with other GalNAc-Ts to initiate the first step of mucin-like O-glycosylation. This involves adding GalNAc residues to the hydroxyl groups on target proteins. When present alone, this modification is known as the Tn antigen (CD175). It can be extended to core-1 and further modified by additional glycosyltransferases, particularly T-synthase and its chaperone Cosmc, serving as a starting point for more complex O-glycan structures^8^. By modifying proteins, GalNT2 influences their physical and chemical properties, including solubility, resistance to proteolysis, and interaction with other biomolecules^9^.

*GalNT2* has been identified in genome-wide association studies (GWASs) as a relevant gene in lipid metabolism that modulates the risk of atherogenic dyslipidemia^10,11^. Aberrant glycosylation, a characteristic of disease and cancer progression, affects a wide range of signaling pathways to promote disease onset and progression. In the context of malignant transformation, altered O-glycosylation, particularly in the form of truncated O-glycans such as Tn and sialyl-Tn antigen (STn, CD175s), contributes to an aggressive and metastatic phenotype^12^. This phenomenon has been notably observed in pancreatic ductal adenocarcinoma^13,14^. Copy number variations (CNVs) affecting GALNT2 are commonly found in various cancers, whereas gene amplifications are more common than deletions. Compared with low GALNT2 expression, high GALNT2 expression was correlated with significantly shorter overall survival in patients with PDAC (*P* = 0.011) (Supplementary Fig. 1).

Additionally, genetic knockout of Cosmc in the exocrine pancreas of mice has been shown to cause the expression of truncated O-glycans (Tn antigen), resulting in exocrine pancreatic insufficiency, reduced digestive enzyme activity, and diabetes^15^.

To gain deeper insight into the physiological role and overexpression of GalNT2 in the pancreas, we generated a conditionally transgenic GalNT2 mouse line to characterize the glycosylation-dependent phenotype. We anticipate that our work will enhance the understanding of the previously underexplored effects mediated by GalNT2 and may inform potential future translational applications.

## Materials & methods

### Conditional transgenic GalNT2 mice

The GalNT2 coding sequence (Gene ID: 2590) was introduced within the Rosa26 locus via homologous recombination in embryonic stem cells. A floxed (lox511 flanked) transcriptional STOP cassette was incorporated between the GalNT2 coding sequence and the CAG promoter to allow the expression of the resulting transgene to be dependent upon Cre recombinase. This mouse strain was developed by GenOway (Lyon, France). Homozygous floxed GalNT2 mice were interbred with either Ptf1a^Cre/+^ (JAX stock #023329) to obtain heterozygous offspring or Ptf1a^Cre/+^;Rosa26^GalNT2/+^ to obtain homozygous Cre-activated offspring. The R26-stop-EYFP mutant mice have a loxP-flanked stop sequence followed by the enhanced yellow fluorescent protein (EYFP) gene inserted into the Gt(ROSA)26Sor locus (Jackson Laboratory; JAX stock #006148). These mice were used for monitoring Cre expression in the pancreas and tracing the lineage of these cells. The genetic background of all the mice used was C57BL/6J (Jackson Laboratory). The mice used in this study were bred at the research animal facility of the University Medical Center Hamburg-Eppendorf. The mice were housed under a 12–12 h light–dark cycle with constant temperature and food and water provided ad libitum. The mice were euthanized by CO_2_ asphyxia and subsequent cervical dislocation. Genotyping was performed via the Kappa Mouse Genotyping Hot Start Kit (PeqLab, Erlangen, Germany). The genotyping primers used were R26-GalNT2-F 5‘-AAGACGAAAAGGGCAAGCATCTTCC-3‘, R26-GalNT2-R 5‘-GCAGTGAGAAGAGTACCACCATGAGTCC-3‘, R26-WT-F 5‘-CAATACCTTTCTGGGAGTTCTCTGC-3‘, R26-WT-R 5‘-CTGCATAAAACCCCAGATGACTACC-3‘, Cre-F 5’-ACCAGCCAGCTATCAACTCG-3’, Cre-R 5’-TTACATTGGTCCAGCCACC-3’, EYFP-F 5’-AGGGCGAGGAGCTGTTCA-3’, and EYFP-R 5’-TGAAGTCGATGCCCTTCAG-3’. All experimental procedures were approved by the Institutional Animal Care Committee and the local animal ethical committee. All methods were performed in accordance with relevant guidelines and regulations and in accordance with ARRIVE guidelines.

### IHC and IF staining

These experiments were performed as we previously described^15,16^. For immunohistochemistry, antibodies against lipase (Cel) (ab79131; Abcam), insulin (8138; CST, Beverly, MA, USA), GalNT2 (PA5-21541, Thermo Fisher Scientific, Grand Island, NY, USA), perilipin (#9349, CST), glucagon (#2760, CST), GFP/YFP (ab290, Abcam) and PNA-fluorescein (FL-1071; Vector Laboratories) were used at a dilution of 1:100. Next, 10 µg/ml biotinylated Vicia villosa lectin (VVA) (B-1235; Vector Laboratories, Burlingame, CA, USA), 10 µg/ml biotinylated peanut agglutinin (PNA) (B-1075; Vector Laboratories) or 10 µg/ml biotinylated Sambucus nigra agglutinin (SNA) (B-1305; Vector Laboratories) complexed with 1 µg of streptavidin-HRP (21126; Pierce, Thermo Fisher Scientific, Grand Island, NY, USA) was used.

### Immunoblotting and neuraminidase digestion

For Western blotting, antibodies directed against GalNT2 (PA5-21541; Invitrogen), fatty acid synthase FAS (#3180; Cell Signaling Technology (CST), Massachusetts, USA), acetyl-CoA carboxylase ACCα (#3676; CST), perilipin (#9349; CST), C/EBPα (#8178; CST), HSP40 (#4871; CST), HSP60 (#12165; CST), HSP90 (#4877; CST), BIP (#3177; CST), PDI (#3501; CST), IRE1α (#3294; CST), calnexin (#2679; CST), SUMO1 (#4930; CST), SUMO2,3 (#4971; CST), and ubiquitin (#3936; CST) were used. For hydrolysis of α2–3-, α2–6-, and α2–8-linked sialic acid residues, 10 µg of total protein was treated with α2–3,6,8-neuraminidase A (P0722; NEB) for 20 min at 37 °C. Next, 10 µg/ml biotinylated PNA (B-1075; Vector Laboratories) complexed with 1 µg of streptavidin-HRP (21126; Pierce, Thermo Fisher Scientific, Grand Island, NY, USA) was used for the detection of nonsialylated core-1. Ponceau S solution was used according to the manufacturer’s specifications (Serva, Heidelberg, Germany).

### Lectin ELISA

Pancreatic lysates (4 µg total protein per well) from WT and GalNT2-TG het mice at 4 and 8 weeks of age (*n* = 3 per group) were coated for 2 h at 4 °C on a MaxiSorp 96-well plate (NUNC). Glycosylation profiles were compared using biotinylated lectins (Vector Laboratories) VVA (B-1235), PNA (B-1075), ConA (B-1005), and SNA (B-1305). As a background control, the lysates were treated with Protein Deglycosylation Mix II (NEB, #P6044L). Carbo-free blocking solution (Vector Laboratories) was diluted in HBSS, and 100 µl per well was used to block for 1 h. Biotinylated lectins were complexed with streptavidin-HRP (Thermo Scientific) in HBSS (Gibco) for 1 h, followed by binding for 2 h. The wells were washed three times with HBSS + 0.05% Tween. ABTS (Roche) was used as the substrate, and the absorbance was measured after 20 min at 405 nm via a FluoStar Omega plate reader (BMG Labtech).

### Measurement of fasting blood glucose

Twelve-week-old WT and GalNT2-het mice (*n* = 8 each) were fasted overnight (12 h), and fasting blood glucose was measured with a glucometer (Accu-Chek Aviva).

### PNA lectin pull-down assay

In brief, agarose-bound PNA (AL-1073, Vector Laboratories) was used to enrich nonsialylated core 1-modified proteins, along with their potential interactors, from pancreatic lysates of WT and GalNT2-TG het mice at 7 weeks of age (2 biological replicates per group). A total of 200 µg of protein was incubated with 70 µl of agarose-bound PNA at 4 °C overnight. Following incubation, the agarose beads were washed, and the bound proteins were eluted with 200 mM galactose (pH 3.0) at 65 °C for 30 min. The eluted proteins were subsequently purified from the elution buffer by SDS‒PAGE.

### Tryptic in-gel digestion

In-gel digestion was performed following the methods of Shevchenko et al.^17^. Shrinking and swelling were performed with 100% ACN and 100 mM NH_4_HCO_3_. In-gel reduction was achieved with 10 mM dithiothreitol (dissolved in 100 mM NH_4_HCO_3_). Alkylation was performed with 55 mM iodacetamide (dissolved in 100 mM NH_4_HCO_3_). Proteins in the gel pieces were digested by covering them with a trypsin solution (8 ng/µL sequencing-grade trypsin, dissolved in 50 mM NH_4_HCO_3_) and incubating the mixture at 37 °C overnight. Tryptic peptides were obtained via extraction with 2% FA and 100% ACN. The extract was evaporated. For LC‒MS/MS analysis, samples were dissolved in 20 µL of 0.1% FA.

### LC‒MS/MS analysis

Protein identification (ID) and quantification (LFQ) via analysis of the tryptic peptides by LC‒MS/MS were achieved by injecting the samples onto a nanoliquid chromatography system (Dionex UltiMate 3000 RSLCnano, Thermo Scientific, Bremen, Germany) coupled via electrospray ionization (ESI) to a mass spectrometer (MS) equipped with a quadrupole, a linear trap and an orbitrap (Orbitrap Fusion, Thermo Scientific, Bremen, Germany). The samples were injected (5 µL/min) into a trapping column (Acclaim PepMap µ-precolumn, C18, 300 μm × 5 mm, 5 μm, 100 Ǻ, Thermo Scientific, Bremen, Germany; buffer A: 0.1% FA in HPLC-H_2_O; buffer B: 0.1% FA in ACN) with 2% buffer B. After sample injection, the trapping column was washed for 5 min with 2% buffer B (5 µL/min). The peptides were moved to the separation column (200 nL/min) and separated on this column (Acclaim PepMap 100, C18, 75 μm × 250 mm, 2 μm, 100 Ǻ, Thermo Scientific, Bremen, Germany; 200 nL/min, gradient: 2 − 30% B in 30 min). The spray was formed by a fused-silica emitter (I.D. 10 μm, New Objective, Woburn, USA) at a capillary voltage of 1650 V. Mass spectra were measured in positive ion mode. LC‒MS/MS analysis with the orbitrap Fusion was carried out in data-dependent acquisition mode (DDA) in top speed mode. An HCD collision energy of 28%, an intensity threshold of 2e5 and an isolation width of 1.6 m/z were used. An MS scan was performed every second over a m/z range from 400 to 1500 (resolution of 120000 FWHM at m/z 200; transient length = 256 ms; maximum injection time = 50 ms; AGC target = 2e5). MS/MS spectra were obtained in an ion trap (scan rate = 66 kDa/s; maximum injection time = 200 ms; AGC target = 1e4; underfill ratio of 10%; isolation width of 2 m/z).

### Data analysis

LC‒MS/MS data were processed with Proteome Discoverer 2.4.1.15 (Thermo Scientific, Bremen, Germany). Identification of the proteins from the MS/MS spectra was performed with the search engine Sequest HT via the *Mus musculus* SwissProt database (www.uniprot.org) and a contaminant database. For the searches, the following parameters were applied: precursor mass tolerance: 10 ppm; fragment mass tolerance: 0.2 Da. Two missed cleavages were allowed. Carbamidomethylation of cysteine residues as a fixed modification and oxidation of methionine residues as a variable modification were used for the search. Peptides whose false discovery rate (FDR) was 1% were identified via Percolator. At least two unique peptides per protein were used as a condition for reliable identification. The online tool Venny 2.1 was used for Venn diagram creation^18^, Enrichr was used for gene ontology enrichment analysis^19^ and BioPlanet2019 was used for pathway analysis^20^.

### Statistical analyses

Each experiment was repeated at least twice. Unless otherwise noted, the data are presented as the means ± SEMs, and a two-tailed, unpaired Student’s t test was used to compare two groups of independent samples. *P* < 0.05 was considered statistically significant. Statistical analysis was performed with Prism 9.5.1 software (GraphPad Software, La Jolla, CA, USA).

### Survival data analysis

Survival analyses were performed via the R2: Genomics Analysis and Visualization platform (http://r2.amc.nl). Where indicated, GalNT2 gene expression was stratified by the median expression, or a percentile ranking derived from the R2 platform’s ‘Kaplanscan’ tool was used to find the cutoff with the most significant p value. The dataset was originally published by Yang et al.^21^. and can be found in the NCBI Gene Expression Omnibus (GEO) database (ID: GSE183795).

## Results

### Reduced pancreatic and body weights in GalNT2 heterozygous mice

**Fig. 1:**
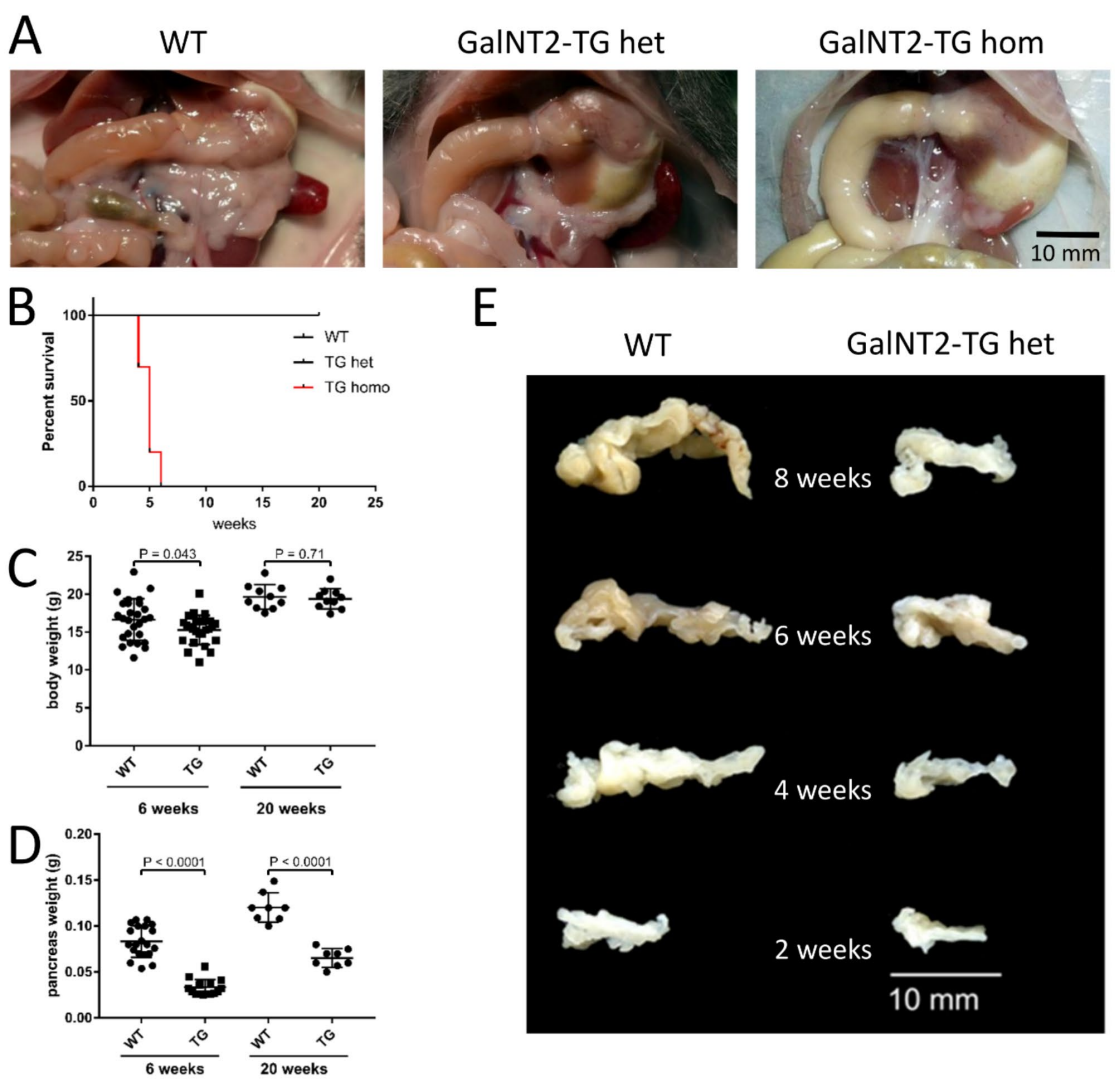
Development of the pancreas and body of WT and GalNT2-TG mice. **(A)** Representative pancreas images from 6-week-old WT, GalNT2-TG het and GalNT2-TG hom mice in situ. The scale bar corresponds to 1 cm. **(B)** Kaplan‒Meier survival curves of WT, GalNT2-TG het, and GalNT2-TG hom mice. **(C)** Body weight analysis of 6-week-old and 20-week-old WT (*n* = 27; *n* = 10) and GalNT2-TG (*n* = 25; *n* = 10) het mice. **(D)** Pancreatic weight analysis of 6-week-old and 20-week-old WT (*n* = 20; *n* = 8) and GalNT2-TG het (*n* = 17; *n* = 8) mice. **(E)** Comparison of formalin-fixed WT and GalNT2-TG pancreata at 2, 4, 6, and 8 weeks of age. The scale bar corresponds to 1 cm.

To investigate the impact of GalNT2 overexpression in the pancreas in vivo, we generated a conditional transgenic GalNT2 mouse and crossed it with the pancreas-specific Cre mouse line Ptf1a^Cre/+^ to generate mice with a heterozygous genotype (GalNT2-TG het). These were then bred with homozygous floxed GalNT2-TG mice to produce homozygous transgenic mice (GalNT2-TG hom). The detection of GalNT2 overexpression in the pancreas is demonstrated in Supplementary Fig. 2 through both Western blot analysis and immunofluorescence (IF) quantification. An in situ comparison of pancreata from wild-type (WT), GalNT2-het, and GalNT2-hom animals at 6 weeks of age revealed a significant reduction in organ size in GalNT2-het animals and almost complete absence in GalNT2-hom animals (Fig. 1A). Mice with the genotypes GalNT2-TG het and hom were viable at birth, with GalNT2-TG hom mice dying between 4 and 6 weeks of age owing to undernutrition (Fig. 1B). Therefore, further phenotypic investigations were conducted on GalNT2-TG het animals. Comparison of body weights between wild-type and GalNT2-TG het mice revealed a slight but significant reduction in the TG group, which was no longer measurable at 20 weeks of age (Fig. 1C). Examination of pancreatic organ weights at 6 and 20 weeks of age revealed significant differences, with the pancreases of GalNT2-TG het animals being significantly smaller and therefore lighter (Fig. 1D). To temporally resolve pancreatic organ development in wild-type and GalNT2-TG het mice between 2 and 8 weeks of age, we present representative organs of each age and genotype in Fig. 1E.

### Histological processing and immunohistochemical staining of pancreatic marker proteins

**Fig. 2:**
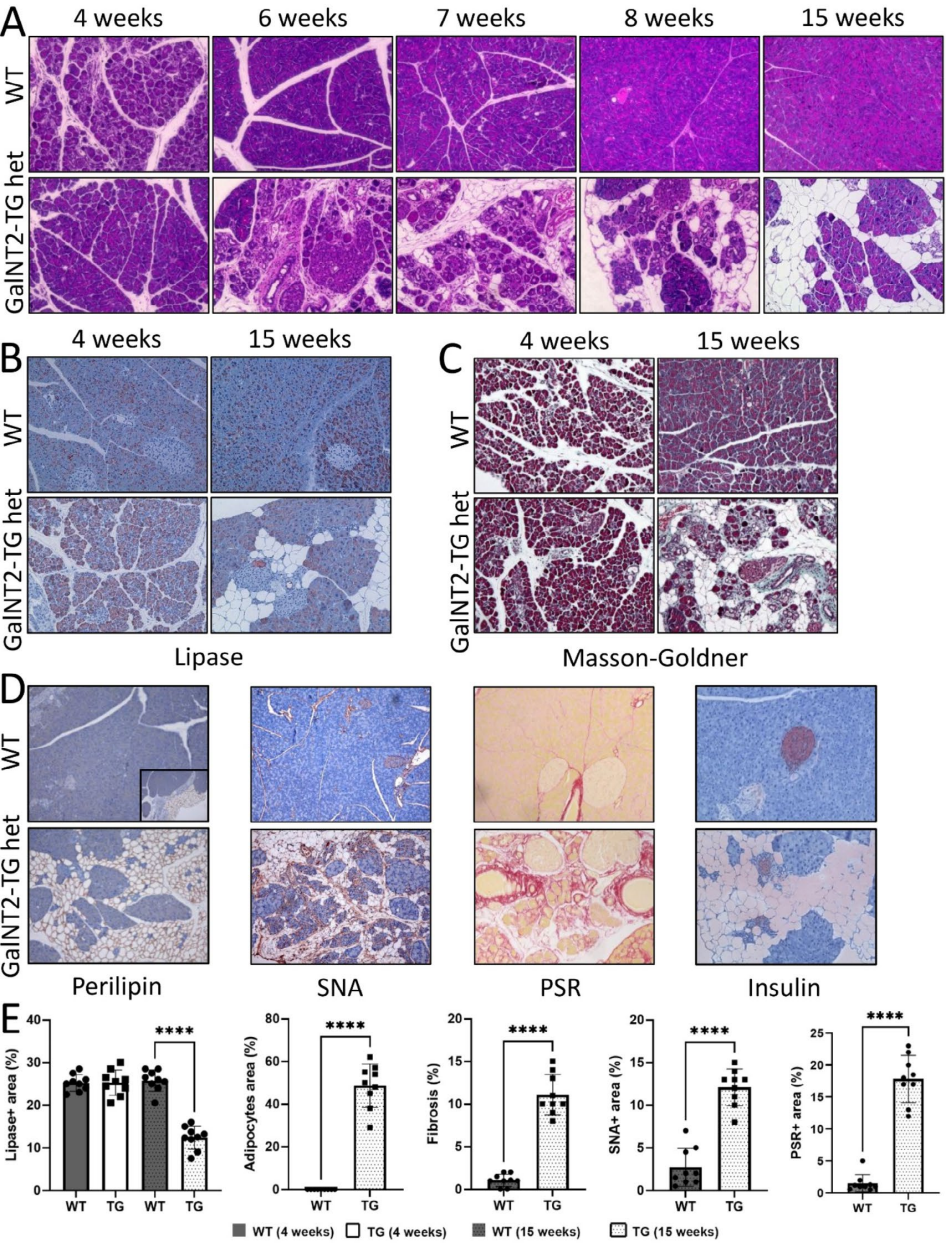
Time-resolved histological staining of pancreatic tissues from WT and GalNT2-TG mice. **(A)** Comparative H&E staining of pancreatic tissues from 4-, 6-, 7-, 8-, and 15-week-old WT and GaltNT2-TG het mice. **(B)** Immunohistochemical (IHC) staining of lipase, a marker of the exocrine pancreas, in murine WT and GalNT2-TG pancreatic tissue sections at 4 and 15 weeks of age. **(C)** Masson–Goldner staining of murine WT and GalNT2-TG pancreatic tissue sections at 4 and 15 weeks of age for the detection of fibrosis. **(D)** Immunohistochemical (IHC) staining of pancreatic, adipocyte, and connective tissue markers in murine WT and GalNT2-TG pancreatic tissue sections. Comparative IHC staining for perilipin, Sambucus nigra agglutinin (SNA), picrosirius red (PSR), and insulin was performed on pancreatic tissue from adult WT and GalNT2-TG heterozygous mice. The anti-perilipin IHC results revealed wild-type visceral adipose tissue located on the pancreas (outlined in a black frame). All micrographs are shown at 200x magnification. **(E)** Quantification of IHC staining as indicated. For each genotype and age group, 3 samples were evaluated, with 3 IHC sections analyzed per sample. **** corresponds to unpaired t test, *P* < 0.0001.

To investigate GalNT2-mediated changes in the pancreas over time, wild-type (WT) and GalNT2-heterozygous tissues were fixed in formalin and embedded in paraffin (FFPE) at ages 4–15 weeks. The tissue sections were stained with hematoxylin and eosin (H&E). In the WT tissue sections, a normal pancreas was observed, characterized by dense acinar tissue, pancreatic ducts, and occasional islets of Langerhans. In contrast, large translucent areas became clearly visible in GalNT2-heterozygous tissue starting from the seventh week of life (Fig. 2A). This observation indicates progressive tissue remodeling, with a notable turning point occurring around the seventh week postnatally. We subsequently performed immunohistochemical staining for lipase, a marker of the exocrine pancreas, on pancreatic tissue sections from murine WT and GalNT2-TG heterozygous mice at 4 and 15 weeks of age. At 4 weeks, the majority of the tissue in both WT and GalNT2-TG heterozygous sections exhibited positive staining for lipase. However, in GalNT2-heterozygous tissues from 15-week-old animals, a significant reduction in the number of acinar cells was observed (Fig. 2B). Furthermore, Masson-Goldner staining revealed fibrosis exclusively in tissues from 15-week-old GalNT2-TG heterozygous animals (Fig. 2C). Adipocytes were detected via perilipin-specific staining. While the WT pancreas was negative for perilipin, the adjacent adipose tissue was positive. In contrast, GalNT2-heterozygous pancreata showed significant perilipin positivity. Sambucus nigra agglutinin (SNA) staining revealed clear positivity in tissues from 15-week-old GalNT2-TG heterozygous animals, similar to the findings from picrosirius red staining. This SNA reactivity was also supported by the results of a lectin ELISA, as shown in Supplementary Fig. 3.

To further assess the presence of connective tissue, type III collagen fibers were stained with picrosirius red (PSR), revealing clear PSR positivity in the remodeled tissue areas of 15-week-old GalNT2-heterozygous pancreata. Additionally, to investigate the integrity of the endocrine pancreas, insulin staining was performed, which labels the pancreatic beta cells within the islets of Langerhans. Insulin-positive islets were observed in GalNT2-heterozygous tissues, although these islets were sometimes not regularly surrounded by acinar cells (Fig. 2D). A quantitative evaluation confirmed that the lipase-positive area was reduced by half in 15-week-old GalNT2-TG heterozygous animals. Correspondingly, there was a proportional increase in the area occupied by adipocytes, reflecting the loss of exocrine pancreatic tissue. Furthermore, quantification of fibrosis, Sambucus nigra agglutinin (SNA), and picrosirius red (PSR) staining revealed a significant increase in these markers in 15-week-old GalNT2-TG tissues (Fig. 2E).

### Increased nuclear density in areas with GalNT2 overexpression leads to acinar-to-adipocyte transdifferentiation

**Fig. 3:**
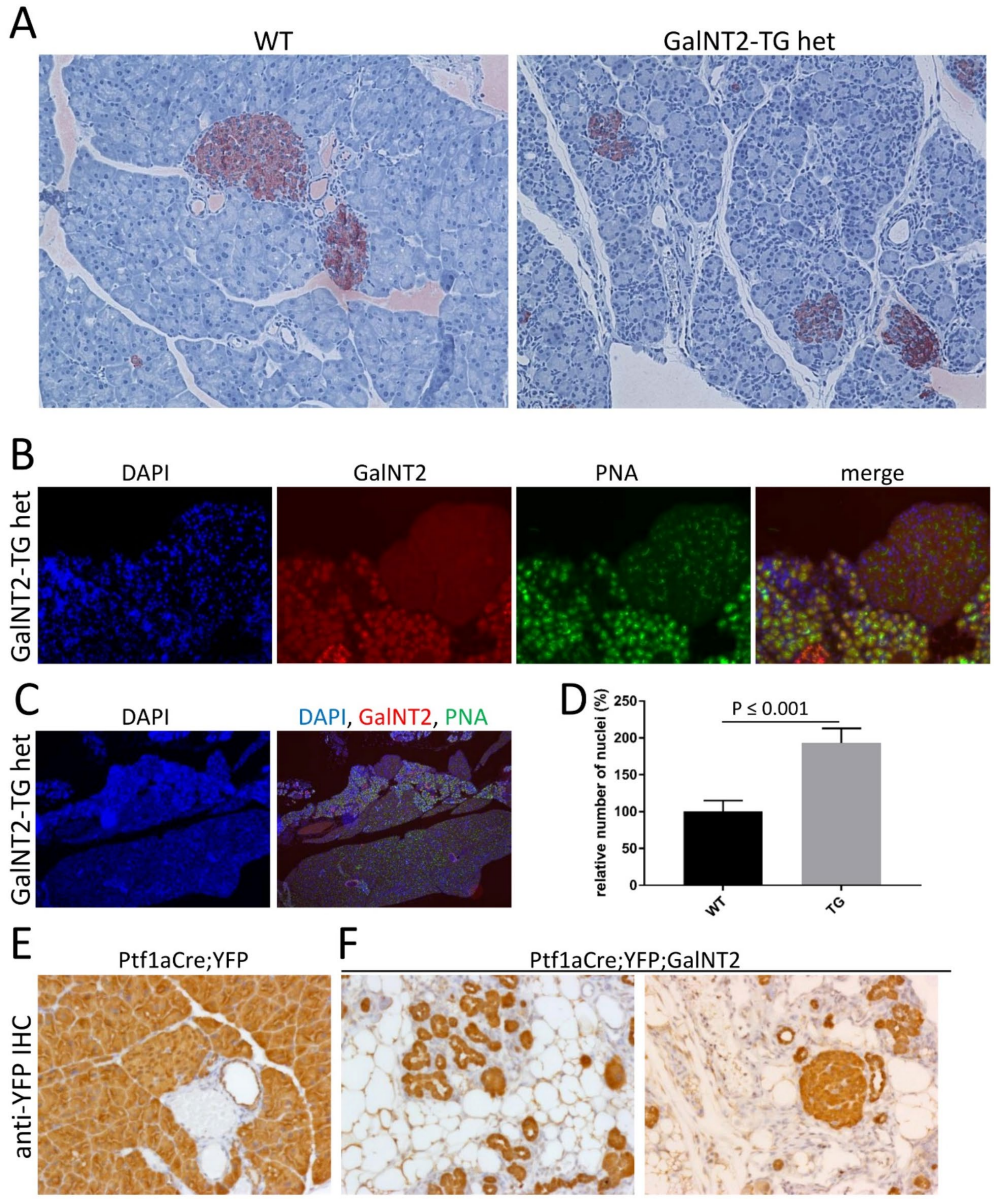
Tissue changes and increased density of cell nuclei in GalNT2-het pancreata. **(A)** Comparative IHC staining of insulin (red) in pancreatic tissues from 4-week-old WT and GaltNT2-TG het mice. **(B)** and **(C)** Comparative IF staining of GalNT2 (red) and core-1 O-glycan (PNA; green) in pancreatic tissue from 4-week-old WT and GaltNT2-TG het mice. DAPI was used to stain the cell nuclei (blue). Magnification in A- B = 200x and C = 40x. **(D)** Relative quantitative assessment of cell nuclei in *n* = 10 fields of view of WT and GalNT2-TG het tissues. **(E**,** F)** IHC staining for YFP in pancreatic tissue derived from 8-week-old reporter strain Ptf1aCre; YFP (corresponding to WT) and Ptf1aCre; YFP; GaltNT2-TG het (corresponding to GalNT2-TG het) mice. Magnification in E and F = 200x.

When stained tissue sections from GalNT2 heterozygous mice aged approximately 5 weeks were analyzed, a significantly increased number of cell nuclei were observed (Fig. 3A). This condition is observed in all GalNT2 heterozygous mice aged 4–6 weeks, always preceding tissue remodeling with adipocytes. Immunofluorescence (IF) analysis with DAPI, GalNT2, and PNA clearly revealed that the increased number of cell nuclei in tissues from GalNT2 heterozygous mice occurred exclusively in areas expressing the transgene (Fig. 3B). Under low-magnification IF, the densely packed cell nuclei (blue) were even more prominent (Fig. 3C). Relative quantification of cell nuclei between WT and GalNT2-TG het pancreata revealed statistically significant doubling (Fig. 3D).

To determine the origin of the adipocytes that replace acinar cells in GalNT2-TG het pancreata between 4 and 6 weeks of age, we introduced a conditionally expressed reporter gene, yellow fluorescent protein (YFP). In reference tissues, where YFP is exclusively expressed under the control of the pancreatic transcription factor Ptf1a, all the cells were YFP positive, indicating that they originated from Ptf1a-expressing progenitor cells (Fig. 3E). In tissues from YFP and GalNT2 double transgenic mice, the somata of adipocytes clearly exhibited YFP positivity (Fig. 3F).

### GalNT2 overexpression altered O-Glycosylation in endocrine alpha cells

**Fig. 4:**
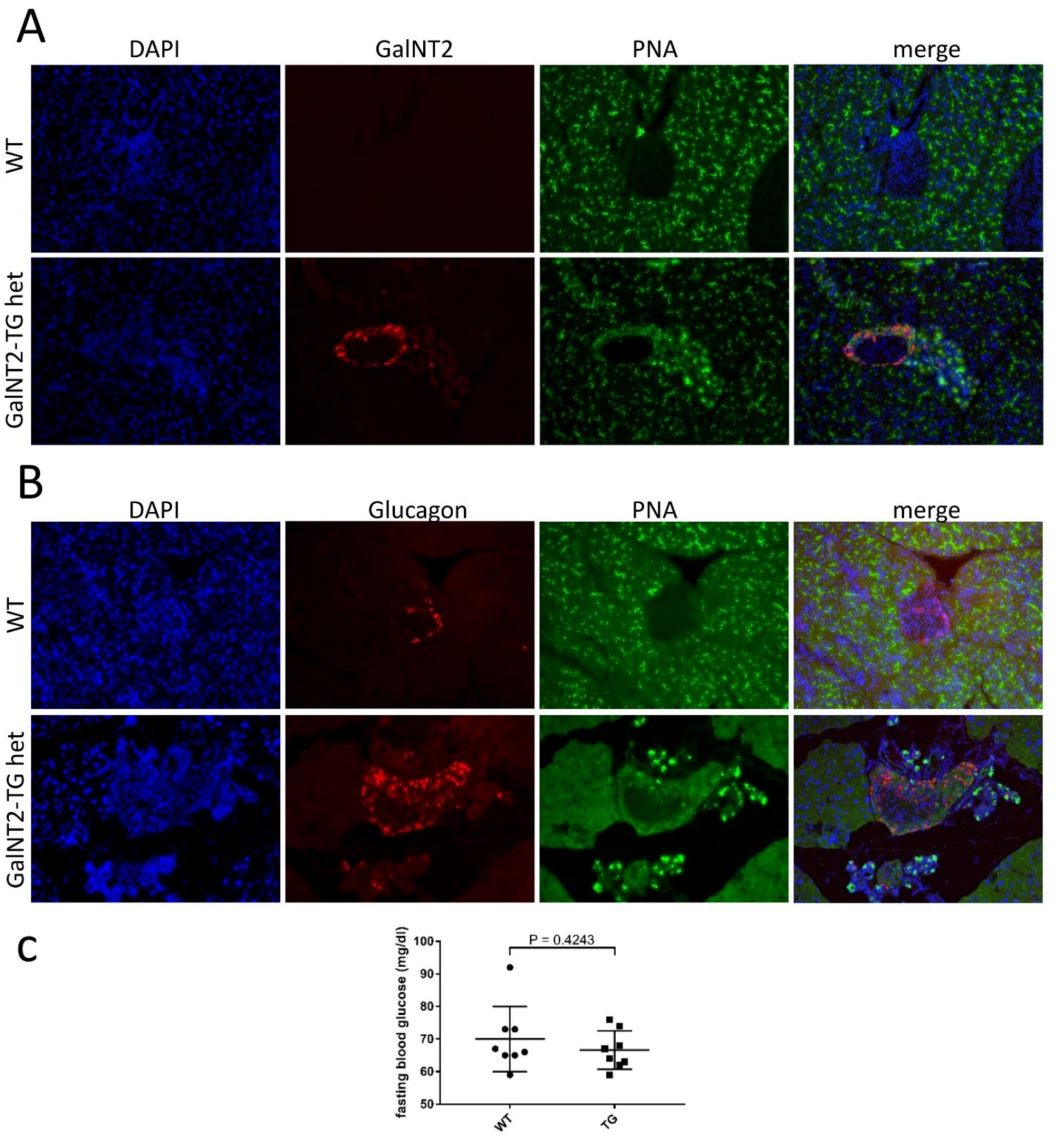
Analysis of GalNT2 localization and associated O-glycosylation in the pancreas of WT and GalNT2-TG het mice. **(A)** Comparative IF staining for GalNT2 (red) and core-1 O-glycans (PNA; green) in pancreatic tissue from 8-week-old WT and GaltNT2-TG het mice. DAPI was used to stain the cell nuclei (blue). **(B)** Comparative IF staining for glucagon (red) and core-1 O-glycans (PNA; green) in pancreatic tissue from 8-week-old WT and GaltNT2-TG het mice. DAPI was used to stain the cell nuclei (blue). Magnification in A and B; 200x. **(C)** Analysis of fasting blood glucose in a cohort of 12-week-old WT and GalNT2-TG het mice (*n* = 8 each).

Next, we investigated the expression of GalNT2 in the pancreas and the impact of its overexpression on O-glycosylation via immunofluorescence staining. For this purpose, we stained tissue sections from WT and GalNT2-TG het mice with a fluorescence-coupled antibody against GalNT2 and with the plant lectin peanut agglutinin (PNA), which binds O-glycosidic core-1 structures (Fig. 4A). The results revealed weak expression of GalNT2 in WT pancreata (red) and pronounced PNA positivity (green), which was confined to the exocrine pancreas. Interestingly, the overexpression of GalNT2 is clearly visible in the tissue, with the outer cells of the islets of Langerhans also showing positivity. Unlike those of the WT, the transgenic cells within the islets of Langerhans were PNA positive, which was not detectable in the WT (Fig. 4A). The overexpression of GalNT2 also results in increased PNA positivity in acinar cells.

Considering that cells within murine islets of Langerhans are organized with alpha cells forming an outer ring and beta cells located internally, we performed double staining with an antibody against glucagon (red) and PNA (green). This revealed a clear colocalization of glucagon-positive alpha cells with altered O-glycosylation (Fig. 4B). This finding prompted the question of whether differential O-glycosylation of alpha cells in GalNT2 in pancreata leads to altered blood glucose regulation. To address this, we measured the fasting blood glucose levels of eight WT and eight GalNT2 het mice at 12 weeks of age. However, no significant difference (*p* = 0.4243) was observed (Fig. 4C).

### Enrichment of Core-1-Modified pancreatic proteins and proteome analysis

**Fig. 5:**
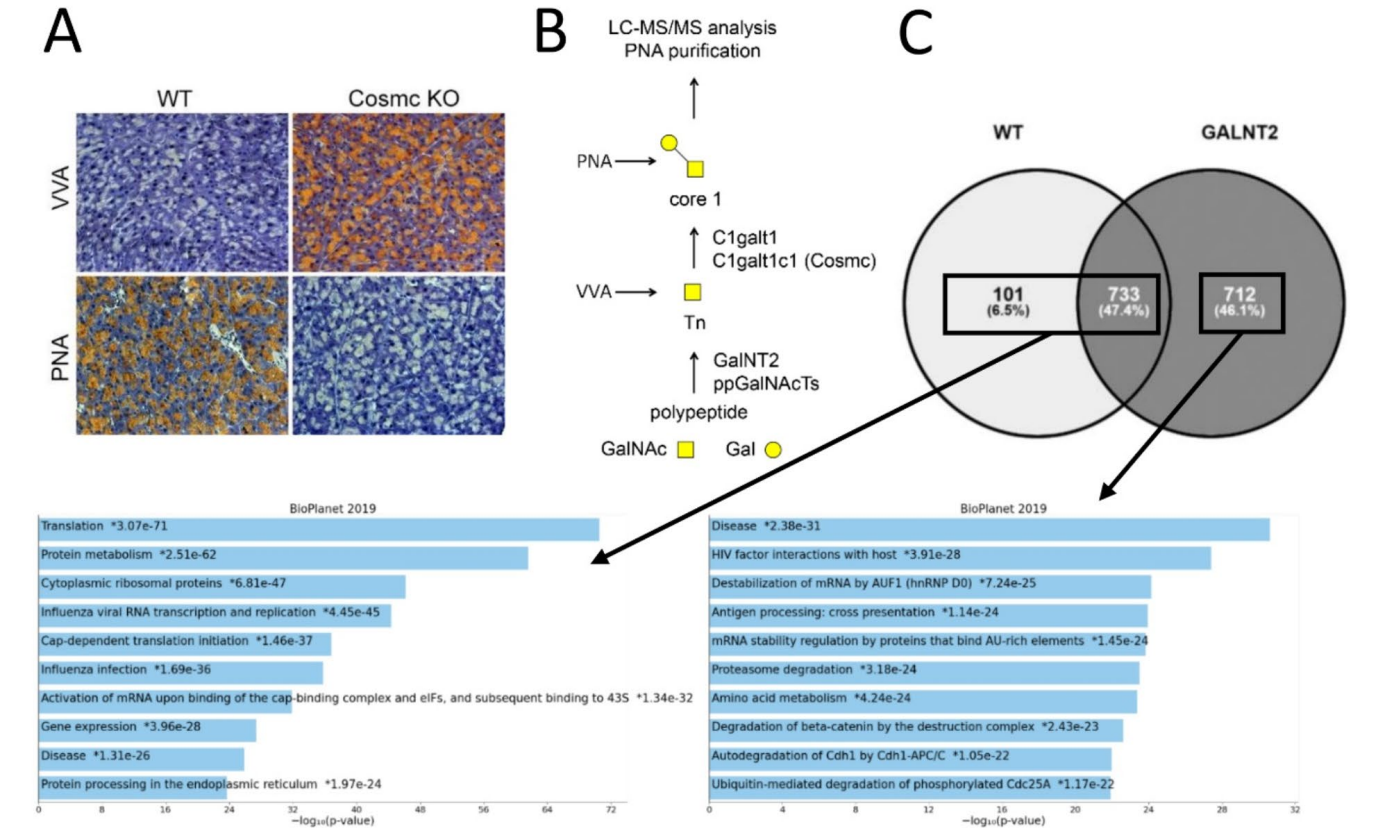
LC‒MS/MS analysis of PNA-purified proteins derived from the pancreas of WT and GalNT2-TG mice. **(A)** Comparative staining of Tn antigen (VVA) and core-1 (PNA) in tissue sections of adult WT and Cosmc-KO murine pancreata. The magnification is 200x. **(B)** Biosynthesis pathway of O-glycans. Starting from a polypeptide, selected serines and tyrosines are modified by GalNT2 (and other ppGalNAcTs) with O-GalNAc (Tn antigen). This glycan is elongated by T-synthase to core-1, which is bound by PNA. **(C)** Venn diagram of identified PNA-purified proteins from WT and GalNT2-TG pancreata (*n* = 2 each) and pathway analysis of WT proteins, including common and exclusively TG-derived proteins.

In humans and mice, O-GalNAc glycans are covalently attached to serine and threonine residues in the endoplasmic reticulum as posttranslational modifications during protein biosynthesis and are extended in a multistep enzymatic process. GalNT2 and other GalNAc-transferases transfer a GalNAc residue onto recipient proteins. This modification, also known as the Tn antigen, plays a role in various diseases. Through the action of the enzyme T-synthase (C1GALT1) and its chaperone Cosmc (C1GALT1C1), the Tn antigen is extended to the core-1 structure (Fig. 5A). Interestingly, the core-1 structure is abundant in WT pancreata and can be detected or enriched for glycoprotein analysis via the PNA lectin. Neuraminidase digestion of murine pancreatic proteins did not result in increased protein detection, suggesting that these proteins exist in a nonsialylated form in vivo, as shown in Supplementary Fig. 5. If T-synthase is nonfunctional in murine pancreata, O-glycosylation remains at the Tn antigen stage, which can be detected via VVA lectin (Fig. 5B).

To determine which core-1-modified proteins are expressed in WT and GalNT2 heterozygous pancreata and how they contribute to the phenotype, we enriched PNA core-1-modified proteins and possibly interacting proteins by PNA lectin pull-down and conducted mass spectrometric proteome analysis. The number of identified proteins from the two genotypes, along with their intersection, is illustrated in a Venn diagram (Fig. 5C). This analysis revealed that the overexpression of GalNT2 significantly influences the glycoproteome, resulting in an increased number of identified proteins in the tissues of GalNT2 heterozygous mice.

Classification of the identified proteins into functional groups (gene ontologies) revealed that a substantial proportion of proteins common to both wild-type (WT) and GalNT2 heterozygous tissues, as well as those identified exclusively in WT tissues, are associated with categories such as translation, protein metabolism, and cytoplasmic ribosomal proteins. In contrast, the analysis of proteins identified solely in GalNT2-TG heterozygous tissues revealed their associations with functional groups related to disease, interactions between HIV factors and the host, mRNA destabilization by AUF1 (hnRNP D0), and antigen processing, including cross-presentation, mRNA stability, and proteasome degradation. The detailed proteomics results are provided in Supplementary Table 1 (PNA pull-down).

## Discussion

As previously reported, interpreting the phenotypes observed in cell or animal models with altered expression of glycosylation-related genes, such as polypeptide GalNAc-transferases, poses significant mechanistic challenges^22,23^owing to the large number of differentially modified proteins involved^4,24^. In the context of the pancreas, only a few studies have explored the impact of glycosylation changes on physiological processes. For example, conditional knockout of *Cosmc* (*C1galt1c1*) has been shown to result in the expression of truncated O-glycan structures (Tn and STn antigens) in the exocrine pancreas, producing a phenotype that resembles maturity-onset diabetes of the young 8 (MODY8)^15^.

In the present conditional transgenic mouse model featuring Ptf1a-Cre-driven overexpression of GalNT2, heterozygous alterations in the exocrine pancreas led to atrophy and pancreatic steatosis, which became notably evident at 6–8 weeks of age. In contrast, homozygous overexpression initially allows for normal pancreatic development but subsequently results in organ degradation within the first 4 to 6 weeks postnatally. This progression culminates in severe pancreatic insufficiency, malabsorption, undernutrition, and ultimately a lethal phenotype.

The application of lipase as a marker for the exocrine pancreas revealed, through comparative immunohistochemical (IHC) staining, that both WT and GalNT2-TG heterozygous animals presented identical proportions of lipase-positive areas at 4 weeks of age. At this stage, markers for fibrosis and connective tissue are also negative. However, between 6 and 8 weeks of age, notable densification of cell nuclei in the GalNT2-TG pancreas occurred, with double the number of nuclei detectable in comparable regions. This state, characterized by elevated O-glycosylation rates, as evidenced by PNA binding, correlates with GalNT2 overexpression for up to weeks 6 to 7. This scenario becomes more pronounced as there is a loss of exocrine tissue accompanied by transdifferentiation into adipocytes. Further investigations are necessary to elucidate the precise role of GalNT2 in the transdifferentiation of acinar cells into adipocytes and to assess the site-specific effects of GalNT2 and its overexpression on the O-glycome.

Interestingly, several findings in the literature suggest that different genetic models can lead to similar pancreatic phenotypes. For example, conditional inactivation of *c-Myc* in Pdx1-expressing pancreatic progenitor cells results in a reduced acinar mass and pancreatic hypoplasia. In adult mice, defective acinar cells in the exocrine pancreas eventually transdifferentiate into adipocytes^25^. Comparative analysis of PNA-enriched proteins from wild-type and GalNT2 transgenic mouse pancreata via CHEA transcription factor targets identified MYC as a significant regulator of the affected genes (Supplementary Fig. 2). This finding implies a possible link between *Myc* knockout and the differential glycosylation of Myc target genes, which could lead to similar phenotypes in the pancreas.

Prox1 (proto homeobox protein 1) has also been identified as a key regulator of exocrine pancreatic development^26,27^. Pancreas-specific deletion of Prox1 induces premature differentiation of acinar cells, increased proliferation of ductal cells, and disrupted ductal morphogenesis. While minor changes are observed in islet cells, beta-cell development remains largely unaffected. However, congenital exocrine defects in *Prox1*-KO mice initiate a progressive deterioration process, leading to significant acinar cell loss, lipomatosis, and ductal tissue damage in adult animals^28^.

Moreover, mutations in the *SBDS* gene (SBDS ribosome maturation factor) have been studied in mice to understand pancreatic defects similar to those observed in Shwachman–Diamond syndrome (SDS), the second most common cause of hereditary exocrine pancreatic insufficiency in humans. Over 90% of SDS patients have biallelic loss-of-function mutations in *SBDS*, which are crucial for ribosome function and are classified among the human ribosomopathies^29,30^. Pancreas-specific knockout of the *Sbds*gene in mice reproduces SDS-like phenotypes characterized by a reduced pancreatic mass, fat deposits, and hypoplastic exocrine compartments with few zymogen granules. The absence of SBDS leads to earlier onset of phenotypes and endocrine impairments, accompanied by reduced serum levels of digestive enzymes and overall growth retardation^31^.

While no direct evidence links SBDS with GALNT2, the phenotypes of *Sbds* knockout mice and *GalNT2* transgenic mice exhibit significant parallels. GalNT2 plays a crucial role in the glycosylation of proteins, a modification essential for their stability and function^32,33^. Our pathway analysis (Fig. 5) suggested that a subset of O-glycosylated proteins identified in the pancreas are vital for ribosomal biogenesis. It has been previously reported that cytoplasmic and nuclear proteins, which play crucial roles in ribonuclear and nuclear processes, can undergo O-GalNAc modifications^34^. This finding suggests two plausible scenarios that might explain the GalNT2-TG phenotype: (1) differential O-glycosylation induced by GALNT2 overexpression impacts ribosomal proteins, leading to a ribosomopathy similar to that of SDS, or (2) GALNT2 overexpression disrupts cellular O-glycosylation processes, causing stress-related ribosomal dysfunction.

The role of acinar-to-adipocyte transdifferentiation in these models remains controversial. In a c-Myc mouse model, acinar-to-adipocyte transdifferentiation was implicated in the onset of pancreatic steatosis, as demonstrated via the use of a reporter gene^25^. However, studies involving Prox1-KO mice reported no evidence of transdifferentiation, as pancreatic cells were negative for X-Gal staining and lacked β-galactosidase immunoreactivity, suggesting that these cells did not originate from transdifferentiated pancreatic acinar cells^28^.

Fatty pancreas disease is frequently observed in medical examinations, with a prevalence of approximately 35%, and is significantly associated with metabolic factors. A recent prospective cohort and Mendelian randomization study demonstrated for the first time a causal relationship, indicating that individuals with intrapancreatic fat deposition exceeding 10% have a threefold increased risk of developing pancreatic cancer^35^. Future studies should investigate whether GalNT2 overexpression or alterations in O-glycosylation play a central role in the development of pancreatic ductal adenocarcinoma (PDAC).

## Electronic supplementary material

Below is the link to the electronic supplementary material.

Supplementary Material 1

Supplementary Material 2

## Data Availability

Data is provided within the manuscript or supplementary information files.
