# Supplementary material for: GalNT2-mediated O-glycosylation affects pancreas development and function in mice
Source: Sci Rep. 2024 Nov 30;14:29760. doi: 10.1038/s41598-024-80276-7 (PMC11607353; doi:10.1038/s41598-024-80276-7)
Supplement: Supplementary file 1 — Supplementary Material 1 [file 41598_2024_80276_MOESM1_ESM.docx]

Supplementary Materials

**GalNT2-mediated O-glycosylation affects pancreas development and function in mice**

Baris Mercanoglu, Sissy-Alina Waschkowski, Elena Neuburg, Nina Schraps, Anastasios D. Giannou, Benjamin Dreyer, Sönke Harder, Markus Heine, Christian F. Krebs, Cenap Güngör, Hartmut Schlüter, Nathaniel Melling, Thilo Hackert, Maximilian Bockhorn, Christoph Wagener and Gerrit Wolters-Eisfeld

**This file includes:**

Supplementary Figure 1: Analysis of GALNT2 copy number variations (CNVs) in human cancers and the impact of GALNT2 expression on overall survival in PDAC patients.

Supplementary Figure 2: Detection of GalNT2 overexpression and quantification of Western blot (WB) and immunofluorescence (IF) signals.

Supplementary Figure 3: Investigation of potential glycosylation differences in pancreatic lysates from WT and GalNT2-TG het mice at 4 and 8 weeks of age via lectin-based ELISA.

Supplementary Figure 4: Western blot marker panel targeting key proteins involved in adipogenesis, heat shock response, chaperone function, and protein folding/stability.

Supplementary Figure 5: Investigation of potential sialylated Core 1 O-glycans in the mouse pancreas.

Supplementary Figure 6: Enrichment analysis of O-glycosylated proteins from GalNT2-TG in pancreata after PNA pulldown via EnrichR.

Original / unmodified Western blots

**Supplementary Figure 1**


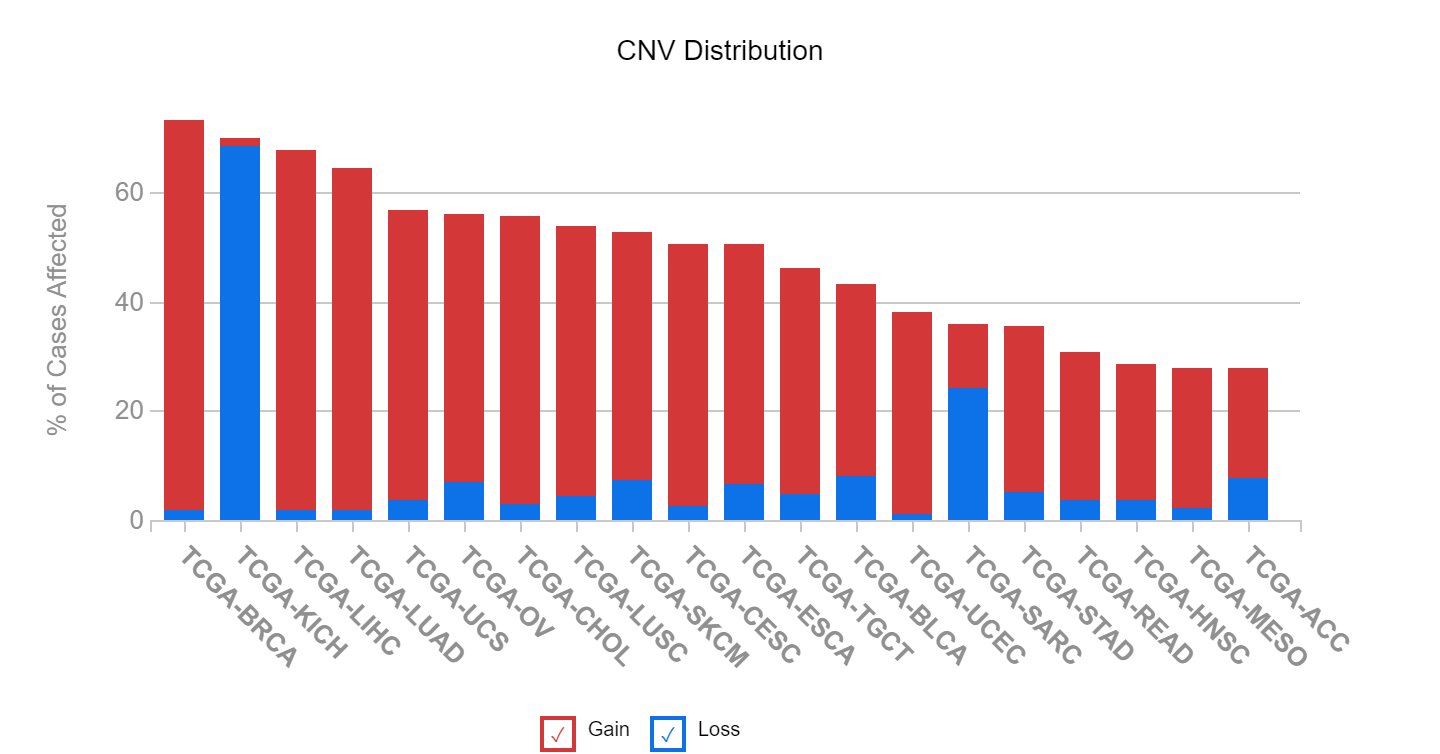


**B**

**A**


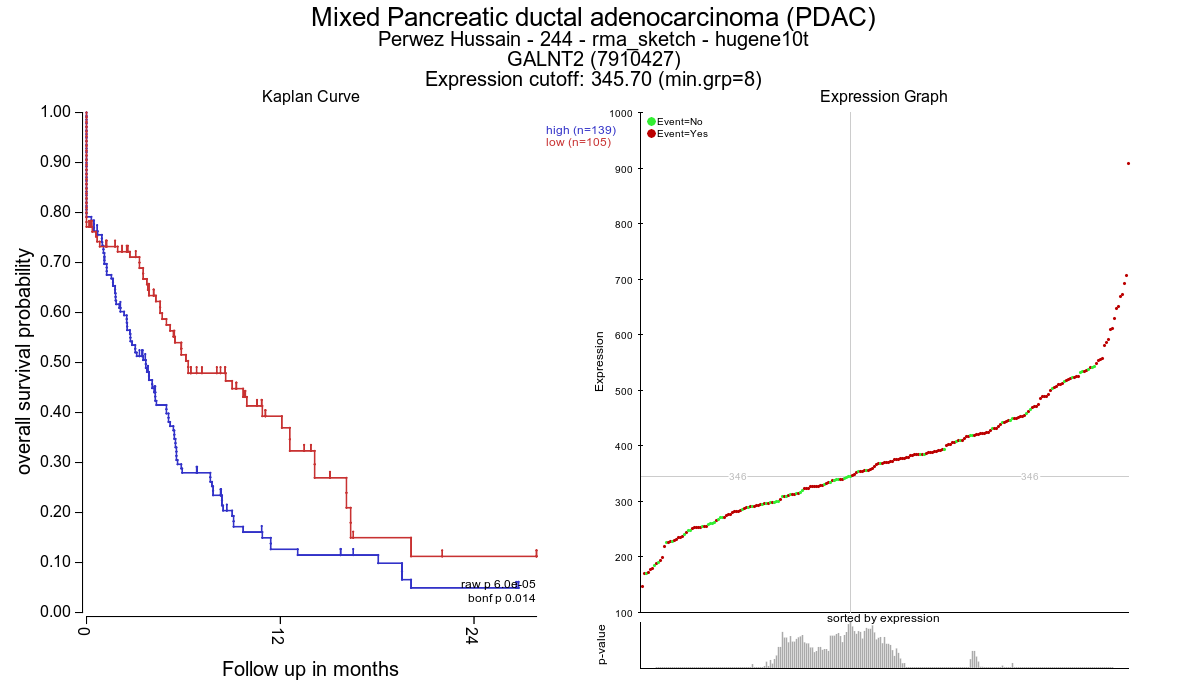


**Supplementary Fig 1. Analysis of GALNT2 copy number variations (CNVs) in human cancers and the impact of *GALNT2* expression on overall survival in patients with PDAC.**

(A) Gains in GALNT2 CNVs are common in human cancers, except for kidney adenomas and adenocarcinomas. (B) High expression levels of GALNT2 in PDAC significantly correlate with decreased overall survival; raw p≤0.0001 and Bonferroni corrected p=0.014.

**Supplementary Figure 2**

**A**

| **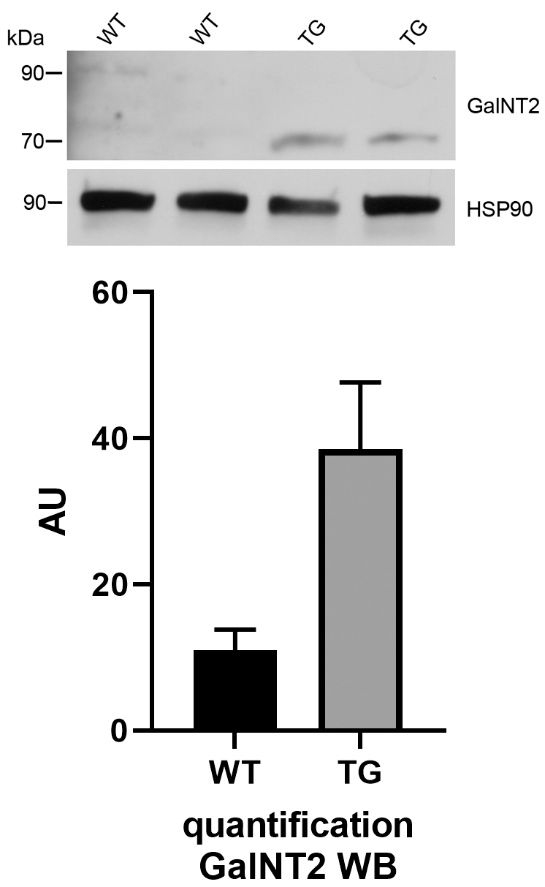** | **B**  **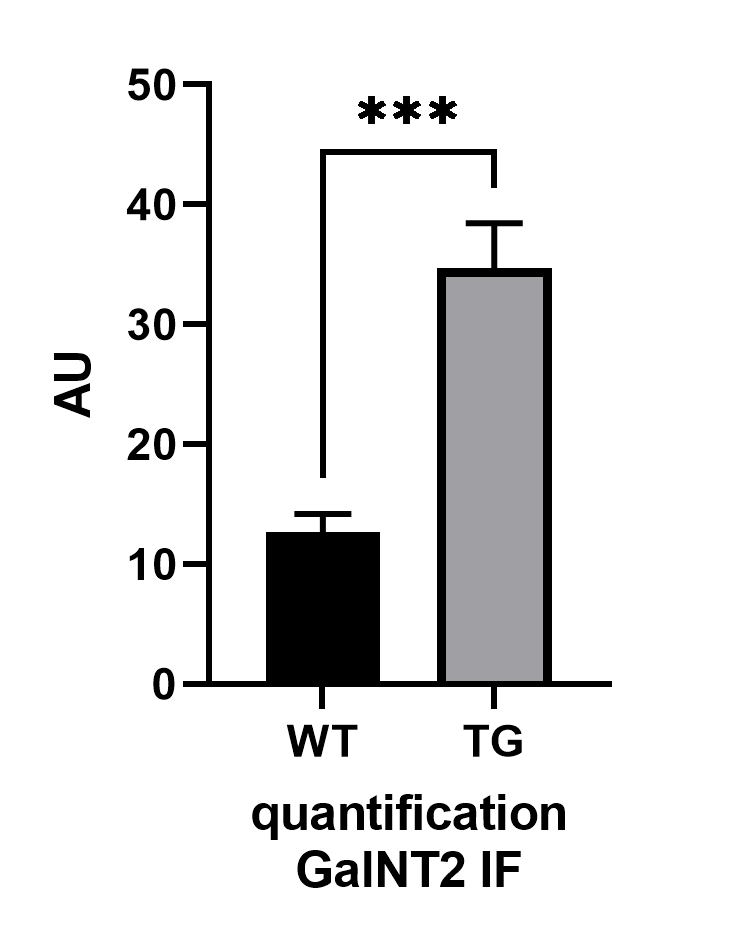** |
| --- | --- |

**Supplementary Fig. 2. Detection of GalNT2 overexpression and quantification of Western blot (WB) and immunofluorescence (IF) signals.**

(A) Western blot analysis of pancreatic lysates from wild-type (WT) and GalNT2-TG heterozygous (het) mice (n=2 per group) with GalNT2 detection. HSP90 was used as a loading control and for quantification. The quantification of GalNT2 bands revealed 4-fold greater expression in GalNT2-TG het mice than in WT mice.

(B) Quantification of IF staining in formalin-fixed, paraffin-embedded (FFPE) pancreatic tissues from WT and GalNT2-TG het mice (n=3 per group) at 4 weeks of age indicating significant 3-fold overexpression of GalNT2 in the GalNT2-TG het group.

**Supplementary Figure 3**

**
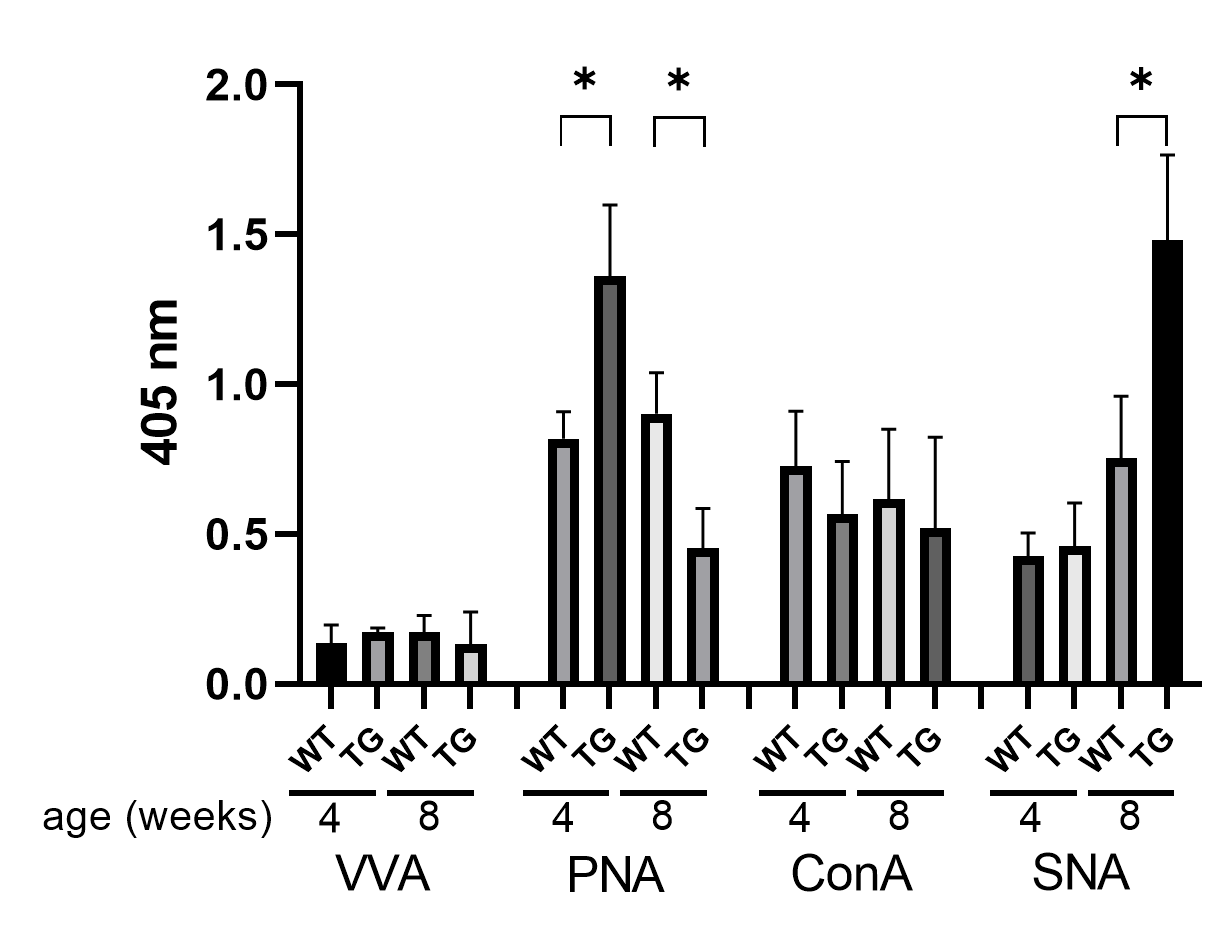
**

**Supplementary Fig 3. Investigation of potential glycosylation differences in pancreatic lysates from WT and GalNT2-TG het mice at 4 and 8 weeks of age via lectin-based ELISA.**

Pancreatic lysates from wild-type (WT) and GalNT2-TG heterozygous (het) mice at 4 and 8 weeks of age (n=3 per group) were coated, and glycosylation profiles were compared via the biotinylated lectins VVA, PNA, ConA, and SNA. VVA, which is specific for O-GalNAc (Tn antigen), showed no binding in the examined samples. PNA, which binds nonsialylated Core 1 structures that are abundantly expressed in the mouse pancreas, was significantly increased in 4-week-old GalNT2-TG mice but notably reduced in 8-week-old mice of the same genotype. N-glycans, detected by ConA, were present in mouse pancreas lysates, but no genotype-dependent changes were observed. SNA, which binds sialylated residues, was significantly increased in the lysates of 8-week-old mice. This result was corroborated by immunohistochemistry (IHC) staining with SNA, as shown in Figure 2.

**Supplementary Figure 4**

**
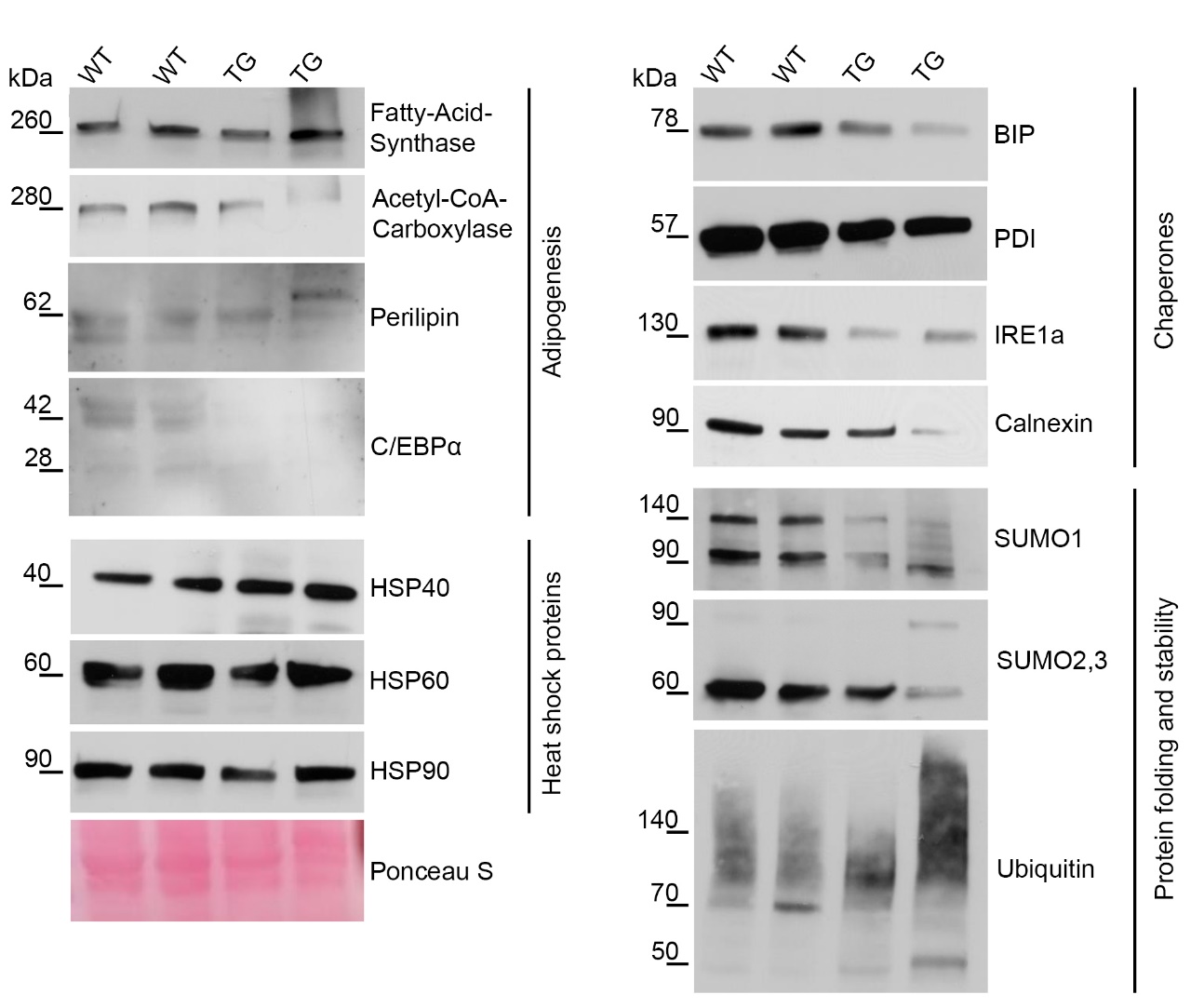
**

**Supplementary Fig. 4. Western blot marker panel targeting key proteins involved in adipogenesis, heat shock response, chaperone function, and protein folding/stability.**

The samples analyzed here were derived from the pancreata of 7-week-old WT and GalNT2-TG het mice (n=2 per group). As shown in Figure 2A, GalNT2-driven tissue remodeling reached a critical point at week 7 and was characterized by pronounced acinar cell death and transdifferentiation into adipocytes. When proteins associated with adipogenesis were examined, the GalNT2-TG het lysates presented reduced expression of C/EBPα, a key regulator of adipocyte differentiation. The detection of perilipin highlights the individual variability in the progression of tissue remodeling, which accounts for the observed differences between GalNT2-TG samples.

The expression of the heat shock proteins HSP40, HSP60, HSP70 (as shown in Fig. 2A), and HSP90 was unchanged, whereas the expression of the chaperones and ER stress markers BIP and IRE1α was reduced in the GalNT2-TG het samples. In animals with elevated perilipin expression, calnexin is notably downregulated. A similar pattern is observed for SUMO1, SUMO2/3, and ubiquitin. Sumoylation by SUMO1 is decreased in GalNT2-TG samples, whereas SUMO2/3 expression increases in a progression-dependent manner, paralleling the changes observed in polyubiquitination.

**Supplementary Figure 5**

**
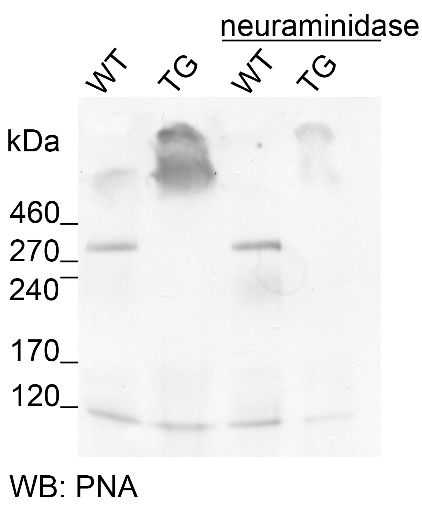
**

**Supplementary Fig. 5. Investigation of potential sialylated Core 1 O-glycans in the mouse pancreas.**

Comparative Western blot analysis of pancreatic lysates from WT and GalNT2-TG heterozygous mice at 7 weeks of age, with and without α2-3,6,8 neuraminidase A treatment. The detection of nonsialylated Core 1 O-glycans was performed via biotinylated peanut agglutinin (PNA).

**Supplementary Figure 6**


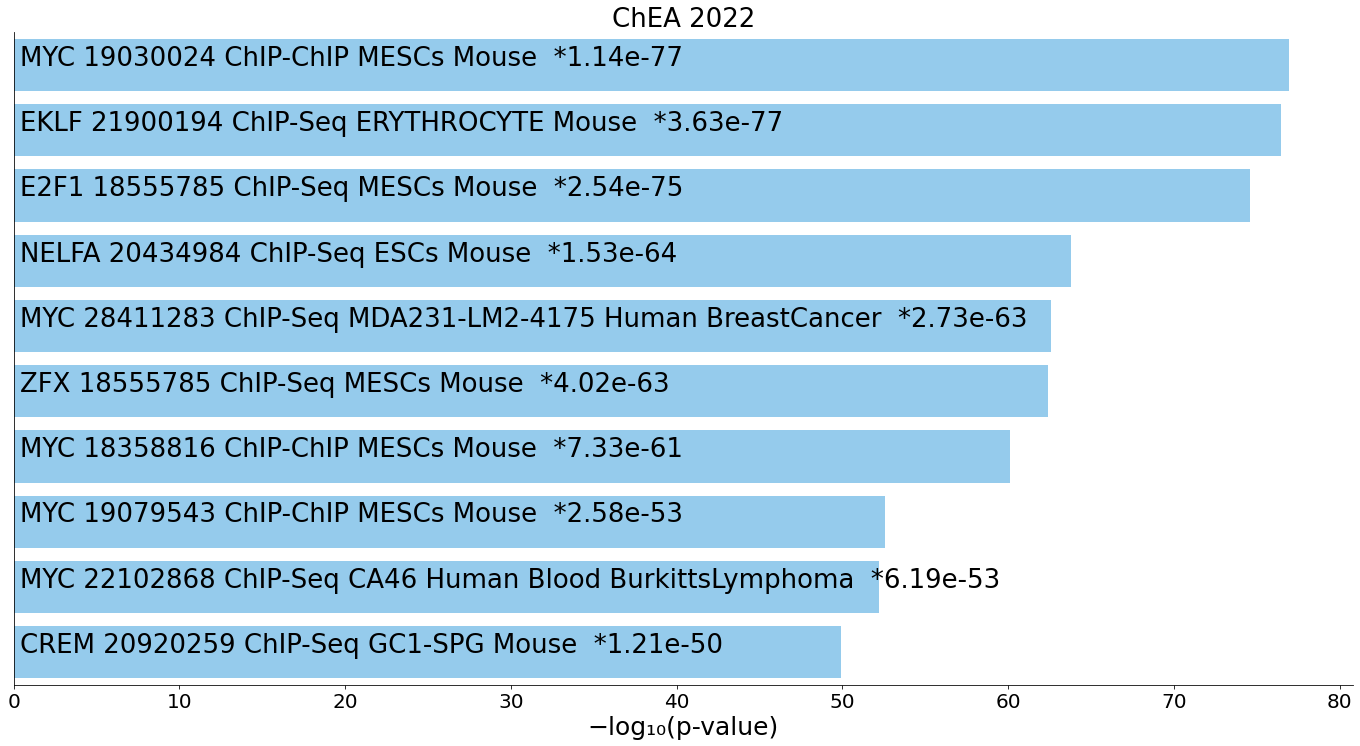


**A**


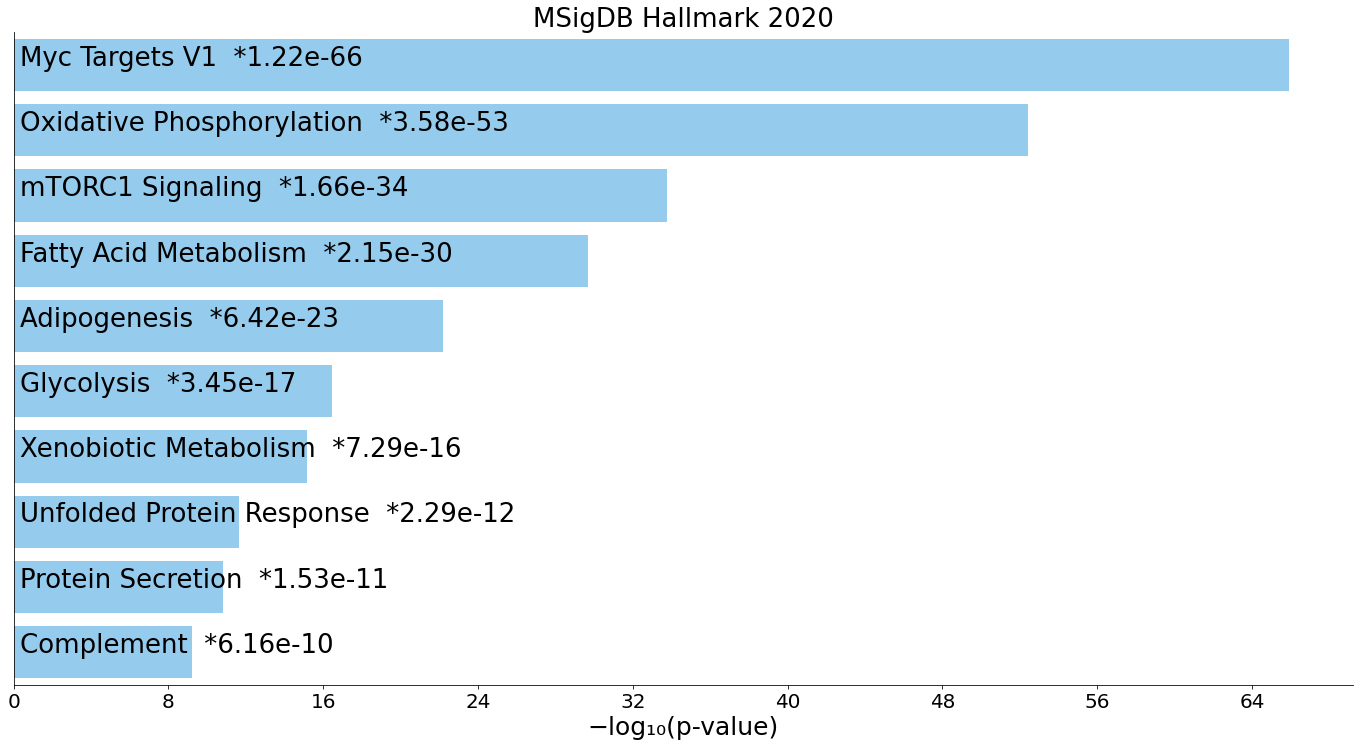


**B**

**Supplementary Fig. 6. Enrichment analysis of O-glycosylated proteins from GalNT2-TG het pancreata after PNA pulldown via EnrichR.**

(A) Identification of potential transcription factors regulating PNA-reactive proteins via ChEA 2022. (B) Identification of the molecular signatures of PNA-reactive proteins isolated from the pancreas of GalNT2-TG patients via the Human Molecular Signatures Database (MSigDB).

**Original / unmodified Western blots:**

| 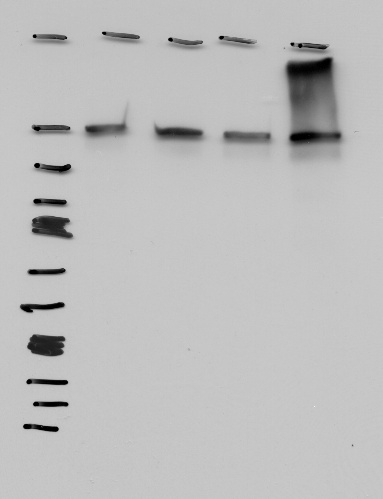  Fatty acid synthase | 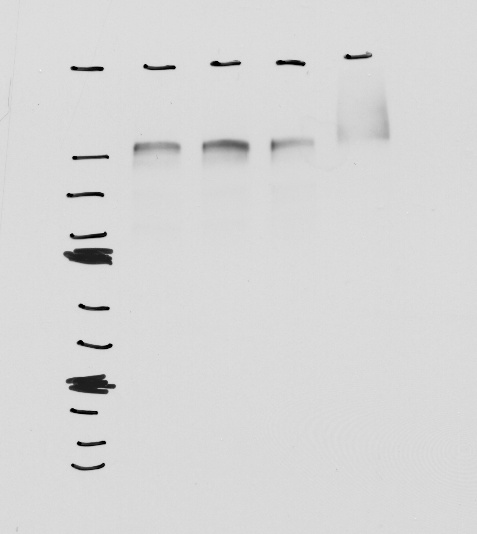  Acetyl-CoA Carboxylase | | 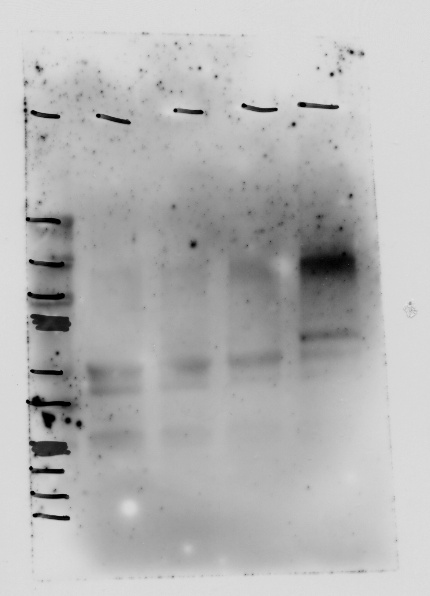  Perilipin |
| --- | --- | --- | --- |
| 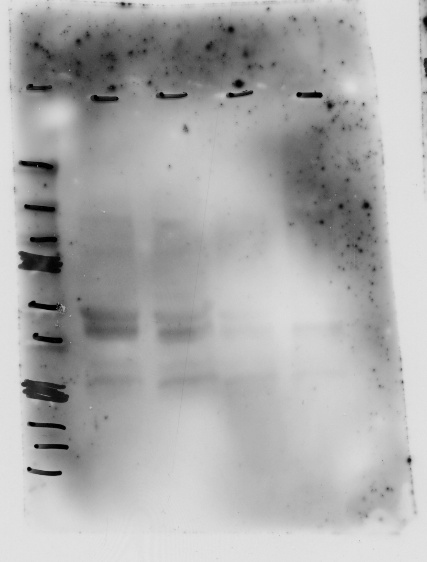  C/EBPa | 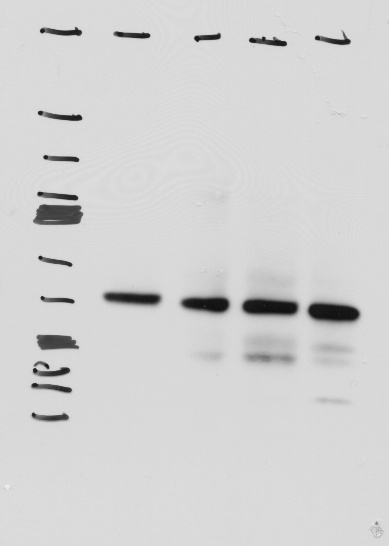  HSP40 | | 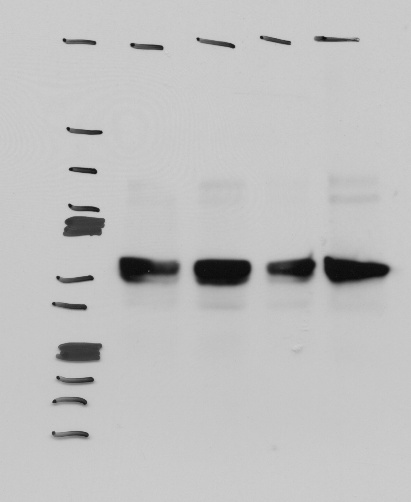  HSP60 |
| 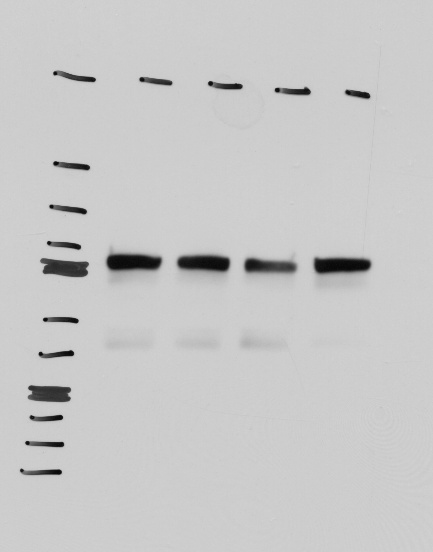  HSP90 | 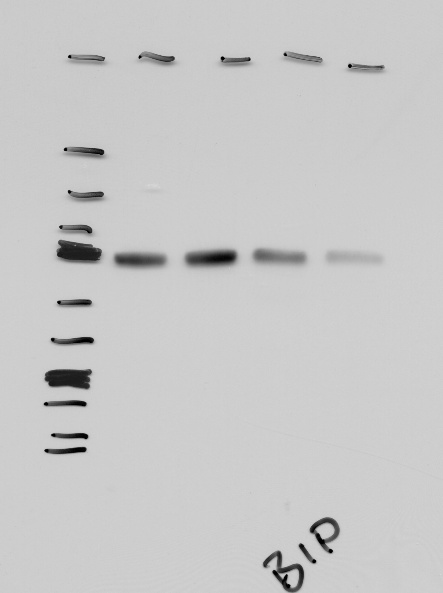  BIP | | 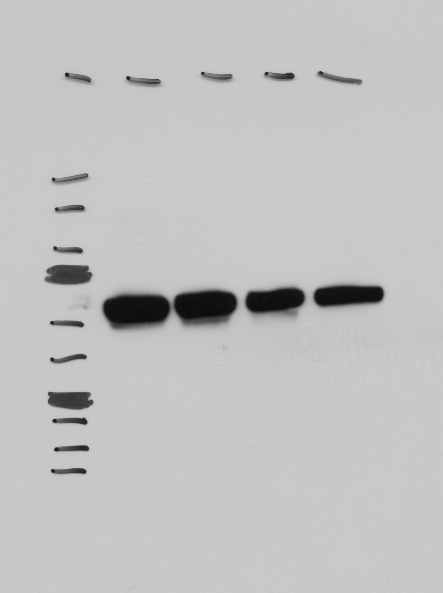  PDI |
| 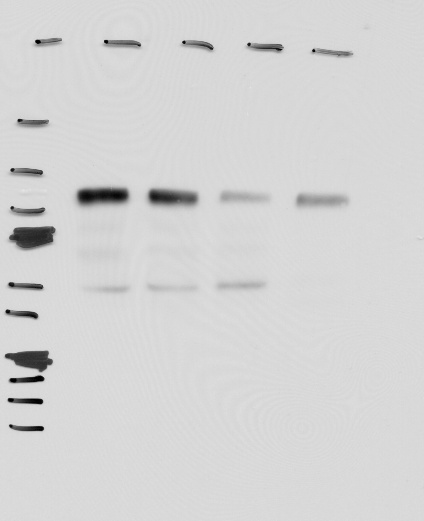  IRE1a | 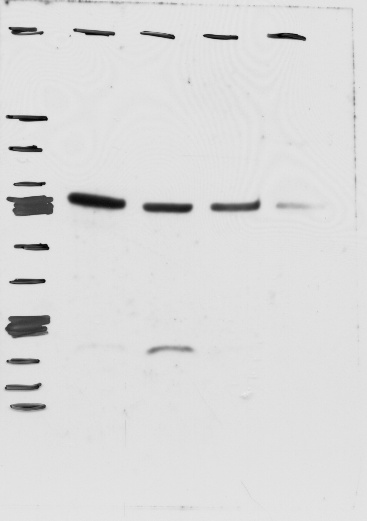 Calnexin | | 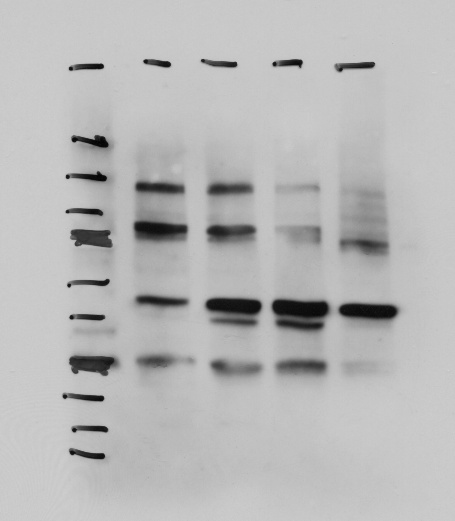  SUMO1 |
| 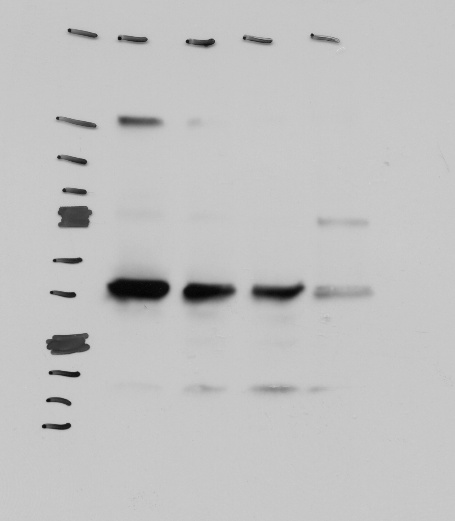  SUMO2,3 | | 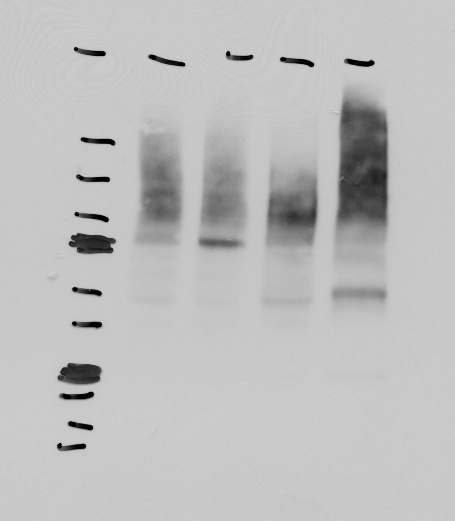  Ubiquitin | 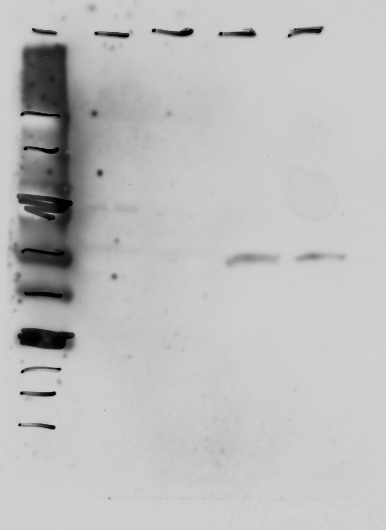  GalNT2 |
